# Meta-analysis of the effect of biofeedback electrical stimulation on postpartum stress urinary incontinence in recent 5 years

**DOI:** 10.1097/MD.0000000000042776

**Published:** 2025-06-13

**Authors:** Jia-Yu Liu, Bin-Han Wang, Ji-Guang Pan, Lu-Wen Zhu

**Affiliations:** a Graduate School, Heilongjiang University of Traditional Chinese Medicine, Harbin, China; b Rehabilitation Center, The Second Affiliated Hospital of Heilongjiang University of Chinese Medicine, Heilongjiang Provincial Key Laboratory of Brain Function and Neurorehabilitation, Harbin, China.

**Keywords:** biofeedback, electrical stimulation, meta-analysis, postpartum stress incontinence

## Abstract

**Background:**

To systematically evaluate the therapeutic effect of biofeedback electrical stimulation on postpartum stress urinary incontinence (PSUI) in the past 5 years.

**Method:**

We searched CNKI, WF, VIP, CBM, Cochrane Library, Web of Randomized controlled trials of biofeedback electrical stimulation for PSUI in Science, Embase, and PubMed databases were searched from July 2019 to July 2024. The outcome indexes included clinical efficacy, pelvic floor muscle strength and ICI-Q-SF score. Literature screening and data extraction of included literatures were conducted independently by 2 reviewers, and literature quality was evaluated according to the standards of the Cochrane Collaboration network. Data analysis was performed using Review Manager 5.3 and stata14.0 software.

**Results:**

A total of 15 literatures were included, with a total of 1362 patients, including 681 in the observation group and 681 in the control group. The results of meta-analysis showed the clinical efficacy of patients in the observation group (OR = 4.11, 95% CI = [3.01–5.60], *P* < .01). Basin muscle strength (OR = 6.01, 95% CI = [3.64–9.94], *P* < .01); International Consultation on Incontinence Questionnaire - Short Form score (SMD = -2.07, 95% CI = [−2.63 to −1.52], *P* < .01);

**Conclusion:**

Biofeedback electrical stimulation has certain curative effect on PSUI, and the therapeutic effect is more significant than that of the control group, which can effectively improve the quality of life of patients with PSUI.

## 1. Introduction

An overextension of the pelvic floor muscles, ligaments, and fascia during pregnancy or childbirth and damage to the pudendal nerve causes postpartum SUI (PSUI). Weak pelvic floor supporting tissue leads to involuntary urine leakage in response to increased abdominal pressure, such as coughing and laughing.^[1]^ Studies have shown that the prevalence of SUI among Chinese adult women is as high as 18.9%; the incidence of maternal urge urinary incontinence and SUI increased from 5.7% and 29.9% during pregnancy to 22.8% and 46.4% at 1 year after delivery and 10.2% and 23.7% at 7 years after delivery, respectively.^[2]^ Although PSUI is not life-threatening, it can cause severe psychological disorders in pregnant women, such as low self-esteem, anxiety, depression, etc. SUI gives rise to these problems that are easily overlooked. They significantly diminish patients’ quality of life and have an impact on their social interaction and participation.^[3]^

Studies have shown^[4]^ that biofeedback electrical stimulation can effectively wake up nerve cells that have lost their conduction function, accelerate the conduction speed of pelvic floor nerve, increase pelvic floor muscle strength, help restore and maintain the stability of urethra and bladder function, and play a specific role in improving vaginal relaxation and urinary and urinary incontinence in patients. By monitoring pelvic floor muscle activity and applying energy electrical stimulation to the damaged perineal nerve, local nerve excitation can be promoted, blood circulation can be accelerated, and muscle contraction ability can be enhanced. Muscle contraction occurs in patients during treatment, and a series of electromyographic activities thus formed can be further converted into images with visual characteristics. Doctors guide pelvic floor muscle functional training based on feedback information, which is conducive to improving the purpose and pertinence of practical training, enabling patients to master the contraction situation by themselves, and improving their subjective initiative.^[5]^ Therefore, biofeedback electrical stimulation can fully play a synergistic role in improving PSUI.

In recent times, the volume of clinical literature on the use of biofeedback electrical stimulation for treating PSUI has been on the rise. This upward trend serves as a clear indication that both clinical and scientific researchers are placing greater emphasis on this particular therapeutic approach. Nevertheless, as per the relevant medical literature retrieved from the evidence-based medicine platform, the existing clinical observation literature in this area is in need of proper systematic analysis. Additionally, more high-quality studies are essential to firmly establish the effectiveness of biofeedback electrical stimulation. Consequently, it is of great necessity to carry out a meta-analysis of the therapeutic effect of biofeedback electrical stimulation on PSUI over the past 5 years. By doing so, we can comprehensively evaluate its clinical efficacy, thereby providing robust evidence-based medical support for the widespread clinical application of biofeedback electrical stimulation in the treatment of PSUI.

## 2. Methods

This review protocol will be carried out and presented in accordance with the guidelines set by the Preferred Reporting Items for Systematic Reviews and Meta-Analysis Protocols (PRISMA–P) statement. It has been registered on the International Prospective Register of Systematic Reviews (PROSPERO), and the assigned trial registration number is CRD42023448170.

### 2.1. Inclusion requirements

The category of the study: the research is a randomized controlled trial (RCT) focusing on the clinical effect of biofeedback electrical stimulation in the treatment of PSUI; study subjects: all patients met the relevant diagnostic criteria in the Guidelines for the Diagnosis and Treatment of Female SUI,^[6]^ and urine pad test was positive and pelvic floor muscle strength assessment ≤ grade 3; intervention approaches: Routine training was provided to the control group (Kegel exercises, Training of pelvic floor muscles, Vaginal dumbbell exercises); The observation group received biofeedback electrical stimulation; Outcome indicators: clinical efficacy,^[7]^ pelvic floor muscle strength, ICI-Q-SF score.

### 2.2. Exclusion standards

Redundant publications; RCT types of studies such as review-type studies, conference-presented content, patents, secondary analyses, and case reports; besides biofeedback electrical stimulation, the observation group incorporated additional therapies that were distinct from those of the control group; previous history of pelvic prolapse, urinary incontinence or neuronal, traumatic and other causes of urinary incontinence; urinary and reproductive system active infection.

### 2.3. Literature retrieval strategy

In this paper, a search was conducted in CNKI, Wanfang (WF), VIP, CBM, the Cochrane Library, and the Web of Science for all RCTs published in Science, Embase and PubMed databases in the last 5 years on the therapeutic effect of biofeedback electrical stimulation on PSUI were searched from July 2019 to July 2024. Chinese search terms: “biofeedback,” “stress incontinence,” “electrical stimulation,” etc. The search strategy was adjusted according to the specific database. English search terms “Electric Stimulation Therapy,” “Biofeedbacks, Psychology,” “Stress Incontinence, Urinary and other search terms can be used for English literature retrieval in the Cochrane Library and PubMed database by combining subject terms with free words. Regarding specific retrieval strategies, the approach used in PubMed serves as an example, which is presented in Table 1.

**Table 1:** Literature screening flow chart.

Process	Search mode
#1	“Stress incontinence, urinary” (Mesh)
#2	Incontinence, urinary stress (Title/Abstract) OR Urinary Stress Incontinence (Title/Abstract) OR stress urinary incontinence (Title/Abstract) OR stress urinary in continence (Title/Abstract) OR stress incontinence (Title/Abstract)
#3	#1 OR #2
#4	“Electric stimulation therapy” (MeSH)
#5	Electrotherapy, interferential current (Title/Abstract) OR interferential current electrotherapy (Title/Abstract) OR interferential current electrotherapy (Title/Abstract)
#6	#4 OR #5
#7	“Biofeedback, psychology” (MeSH)
#8	Physiological feedback, Bogus (Title/Abstract) OR physiological feedback, false (Title/Abstract) OR false physiological feedback (Title/Abstract) OR biofeedback (Title/Abstract) OR psychology biofeedback (Title/Abstract)
#9	#7 OR #8
#10	#3 AND #6 AND #9

### 2.4. Literature screening and data extraction

Two researchers independently carried out literature searches and screenings. Initially, all the literatures retrieved in the preliminary search were imported into the NoteExpress software. By leveraging the software’s function, duplicate literatures were eliminated. Then, through reading titles, abstracts, and keywords, those literatures that were clearly irrelevant were excluded. Subsequently, the full texts of the potentially eligible literatures were read for further screening. The literatures that did not meet the inclusion criteria were removed, and the final set of included literatures was obtained. In case of any disagreements between the 2 researchers, a third-party would be involved to review and make a decision.

The data extracted mainly consist of the author, publication year, sample size, patient age, intervention measures, treatment duration, and outcome indicators. In the event that the information in the article is incomplete, the corresponding author will be contacted.

### 2.5. Literature quality evaluation

The quality of included literatures was evaluated according to the risk bias assessment tools provided by Cochrane. The evaluation included random sequence generation, assignment concealment, blinding of subjects and measures, blinding of outcome evaluators, outcome data integrity, selective reporting, and other bias. According to the bias risk assessment criteria, the risk assessment of the included literature was carried out 1 by 1. The results were classified as “high risk,” “low risk,” or “unclear.” The bias risk bar chart and bias risk summary chart were drawn with the software Review Manager 5.3.

### 2.6. Statistical methods

Statistical analysis was performed using Review Manager 5.3 and Stata14.0 statistical software. For binary variables, the odds ratio (OR) and 95% confidence interval (95%CI) were adopted as the combined effect size indicators. For continuous variables, the standardized mean difference and 95% confidence interval (95%CI) were utilized as the pooled effect size indicators.

The *Q* test and *I*^2^ were employed to evaluate the heterogeneity among the studies. For the included studies with no or low heterogeneity (*I*^2^ < 50%, *P* > .1), the fixed-effect model (FEM) was applied for analysis. However, if the included studies had high heterogeneity (*I*^2^ ≥ 50%, *P *< .1), the random-effects model (REM) was used for analysis. Subgroup analysis was carried out to explore the factors that might contribute to the heterogeneity.

The Stata14.0 software was used to draw a funnel plot and analyze publication bias. The presence of statistical significance was indicated by the *P*-value. When *P* < .05, the difference was regarded as significant; otherwise, there was no significant difference.

## 3. Results

### 3.1. General information on the included literature

**Figure 1.**
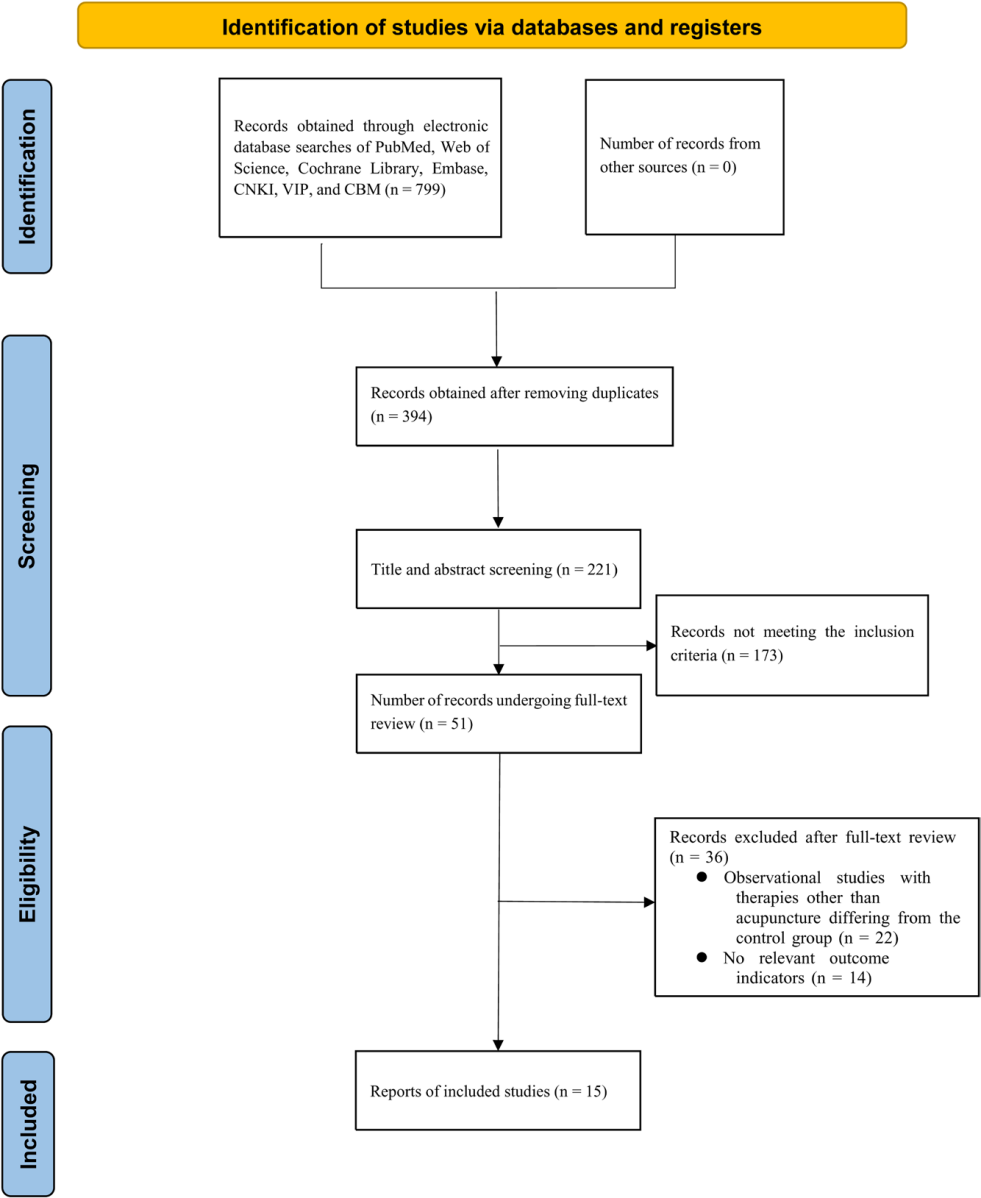
Literature screening flow chart.

A total of 61 RCTs were retrieved, and 7 RCTS were finally included by excluding duplicate papers, checking the title abstracts and reading the full text. The screening flow chart is shown in Figure 1.

### 3.2. Basic features of included studies

In this meta-analysis, 799 literatures were initially examined, and 394 were left after removing duplicate literatures. Further reading the titles, abstracts and full papers excluded reviews, conferences, patents, secondary analyses, case reports, non-RCTs and other literatures that did not meet the inclusion criteria were carried out. Finally, 15 RCTs^[8–21]^ were included, all of which were Chinese literatures. The included publication period was 2019 to 2023, and all were RCTs. There were 466 patients, including 234 in the observation group and 232 in the control group. The intervention measures of 7 literatures^[8–11,13,16,18,21]^ were biofeedback electrical stimulation plus pelvic floor muscle training. Six articles^[11,12,14,15,17,20]^ used biofeedback electrical stimulation and kegel training as intervention measures. In 1 study^,[18]^ biofeedback electrical stimulation and vaginal dumbbell training were used as intervention measures. One^[19]^ paper used biofeedback electrical stimulation alone as an intervention. The basic characteristics of the included literatures are shown in Table 2.

**Table 2:** Basic features of the study were included.

Inclu ded studies	Sample size T/C	Average age (yr)		Interventions		Duration of treatment	Observe metrics
		T	C	T	C		
Yunjie and Xiu^[8]^	45/45	27.8 ± 3.9	27.6 ± 3.2	Biofeedback electric stimulation+Pelvic floor muscle training	Pelvic floor muscle training	Three times a week;8 wk	①③
Liu et al^[9]^	30/30	26.3 ± 3.7	25.7 ± 4.1	Biofeedback electric stimulation+Pelvic floor muscle training	Pelvic floor muscle training	Three times a week;12 wk	①②
Zhou^[10]^	20/20	29.12 ± 7.41	29.58 ± 7.45	Biofeedback electric stimulation+Pelvic floor muscle training	Pelvic floor muscle training	Two times a week;5 wk	①②
Jing et al^[20]^	51/51	29.48 ± 2.53	29.16 ± 2.47	Kegel training+Pelvic floor muscle biofeedback electrical stimulation combined treatment	Kegel training	Two times a week;8 wk	①②
Song et al^[11]^	29/29	43.16 ± 3.29	43.09 ± 3.24	Biofeedback electric stimulation+Pelvic floor muscle training	Pelvic floor muscle training	Seven times a week;4 wk	①②
Zhang et al ^[12]^	49/49	30.61 ± 5.42	30.59 ± 5.36	Kegel training+Electromyography triggered stimulation	Kegel training	Two times a week;8 wk	①③
Jiaqi et al^[13]^	50/50	25. 40 ± 7. 20	25.90 ± 6.50	Biofeedback electric stimulation+Pelvic floor muscle training	Pelvic floor muscle training	–;12 wk	①③
Shi et al^[14]^	70/70	32. 60 ± 4. 12	31.56 ± 4. 68	Electrical stimulation biofeedback therapy+Kegel training+Breathing training+Core strength training	Kegel training+Breathing training+Core strength training	Three times a week;8 wk	①②③
Li et al^[15]^	47/47	29.97 ± 2.13	29.99 ± 2.10	Kegel training+Pelvic floor muscle biofeedback combined therapy	Kegel training	Three times a week;4 wk	①②
Li^[16]^	175/175	25.4 ± 7.8	26.4 ± 6.7	Electrical stimulation biofeedback therapy+Pelvic floor muscle training	Pelvic floor muscle training	Two times a week;12 wk	①②
Niu^[17]^	43/43	28.95 ± 2.75	29.08 ± 2.60	Kegel training+Electromyography triggered stimulation	Kegel training	Two times a week;8 wk	①③
Wang^[18]^	42/42	26.97 ± 2.33	26.43 ± 2.18	Biofeedback electric stimulation+Vaginal dumbbell training	Vaginal dumbbell training	Three times a week;8 wk	①
Aiping and Yuhong^[19]^	30/30	41.12 ± 3.24	35.62 ± 2.87	Pelvic floor biofeedback electrical stimulation	Kegel training	Two times a week;8 wk	①③
Jing et al^[20]^	40/40	-	-	Kegel training+Biofeedback electric stimulation	Kegel training	Three times a week;5 wk	①③
Chen^[21]^	38/38	29.7 ± 0.3	29.6 ± 0.4	Biofeedback electric stimulation+Pelvic floor muscle training	Pelvic floor muscle training	Two times a week;12 wk	①②

① clinical efficacy, ② pelvic muscle strength, ③ ISI-Q-SF score.

ISI-Q-SF = International Consultation on Incontinence Questionnaire - Short Form, T = treatment group, C = control group.

### 3.3. Quality evaluation of included documents

**Figure 2.**
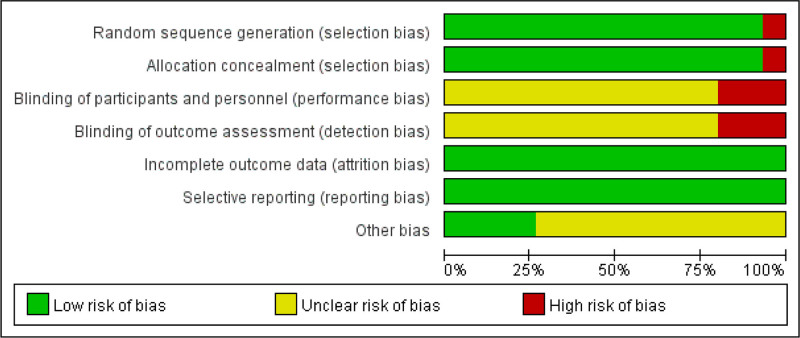
Risk ratio of literature bias (1).

**Figure 3.**
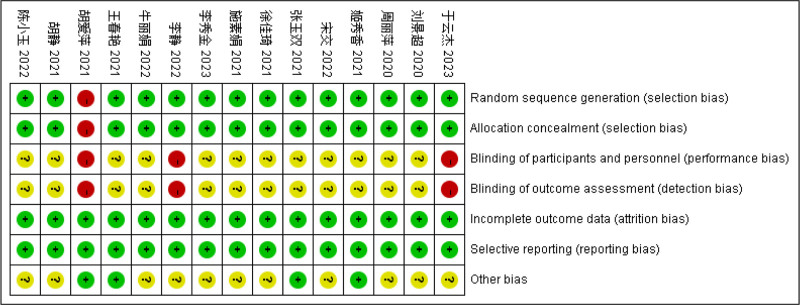
Risk ratio of literature bias (2).

The references incorporated in this study were appraised in accordance with the Cochrane manual. In total, 15 references were included, all of which were in the Chinese language. Among them, 13 references^[9–18,20,21]^ were grouped using the random number table method. One reference^[8]^ made a mention of the randomization method yet failed to describe it in detail, and 1 reference^[19]^ had a high-risk randomized control method. None of the included literature made any reference to assignment concealment and double-blindness. All the study data were complete, and no other biases were stated. The quality evaluation of the included literature is presented in Figures 2 and 3.

Based on the revised Jadad scale, the quality of the included literatures was evaluated. A score ranging from 1 to 3 was considered as low-quality, while a score from 4 to 7 was regarded as high-quality. Please refer to Table 3.

**Table 3:** Literature quality evaluation of revised Jadad scale.

Included studies	Random sequence generation	Allocation concrealment	Blinding	Withdrawal	Total score	Article quality
Yunjie and Xiu^[8]^	1	1	0	1	3	Low quality
Liu et al^[9]^	2	1	0	1	4	High quality
Zhou^[10]^	2	1	0	0	3	Low quality
Jing et al^[20]^	2	1	0	1	4	High quality
Song et al^[11]^	2	1	0	1	4	High quality
Zhang et al^[12]^	2	1	0	2	5	High quality
Jiaqi et al^[13]^	2	1	0	1	4	High quality
Shi et al^[14]^	2	1	0	0	3	Low quality
Li et al^[15]^	2	1	0	1	4	High quality
Li^[16]^	2	2	0	0	4	High quality
Niu^[17]^	2	1	0	1	4	High quality
Wang^[18]^	2	1	0	1	4	High quality
Aiping and Yuhong^[19]^	0	0	0	1	1	Low quality
Jing et al ^[20]^	2	1	0	0	3	Low quality
Chen^[21]^	2	1	0	0	3	Low quality

### 3.4. Results of meta-analysis

#### 3.4.1. Clinical efficacy

**Figure 4.**
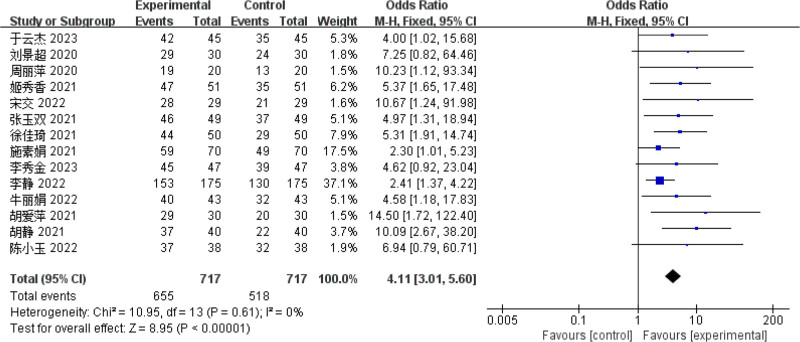
Forest map of clinical efficacy meta-analysis.

Fourteen literatures^[8–17,19–21]^ reported clinical efficacy. Results of the heterogeneity test indicated that multiple studies exhibited homogeneity (*P* = .61; *I*^2^ = 0%), consequently, a FEM was applied to compute the combined effect size. The findings demonstrated that the clinical efficacy of the observation group was markedly superior to that of the control group, and the difference was statistically significant (OR = 4.11, 95% CI = [3.01, 5.60], *P *< .01), indicating that biofeedback electrical stimulation had a significant effect on the clinical efficacy of PSUI (see Fig. 4).

#### 3.4.2. Pelvic floor strength

**Figure 5.**
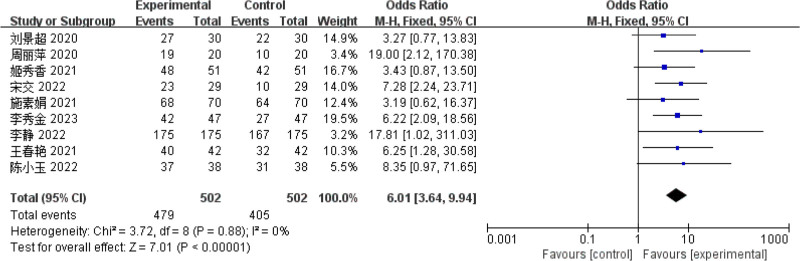
Forest map of pelvic floor muscle strength meta-analysis.

Nine papers^[9–11,14–16,18,21]^ reported pelvic floor muscle strength, and the heterogeneity test results implied that the studies were homogeneous (*P* = .88; *I*^2^ = 0%), therefore, a FEM was utilized for the meta-analysis. The outcomes demonstrated that the improvement in swallowing ability within the observation group was remarkably better compared to that in the control group, and the difference was statistically significant (OR = 6.01, 95% CI = [3.64, 9.94], *P* < .01), indicating that biofeedback electrical stimulation had a significant effect on the treatment of PSUI (Fig. 5).

#### 3.4.3. ISI-Q-SF rating scale

**Figure 6.**
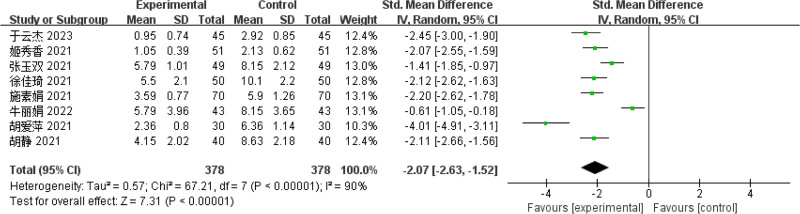
Forest map of ISI-Q-SF meta-analysis. ISI-Q-SF = International Consultation on Incontinence Questionnaire - Short Form.

Eight articles^[8,11–14,17,19,20]^ reported the International Consultation on Incontinence Questionnaire - Short Form (ISI-Q-SF) score, and the heterogeneity test indicated that there was heterogeneity among the studies (*P* = .01; *I*^2^ = 90%), so the REM was used to calculate the combined effect size. The final results showed that the decrease in ISI-Q-SF score in The performance of the observation group was significantly superior to that of the control group, and the difference reached statistical significance (SMD = −2.07, 95% CI = [−2.63 to −1.52], *P* < .01), indicating that biofeedback electrical stimulation had a significant effect on the improvement of ISI-Q-SF in PSUI (see Fig. 6).

**Figure 7.**
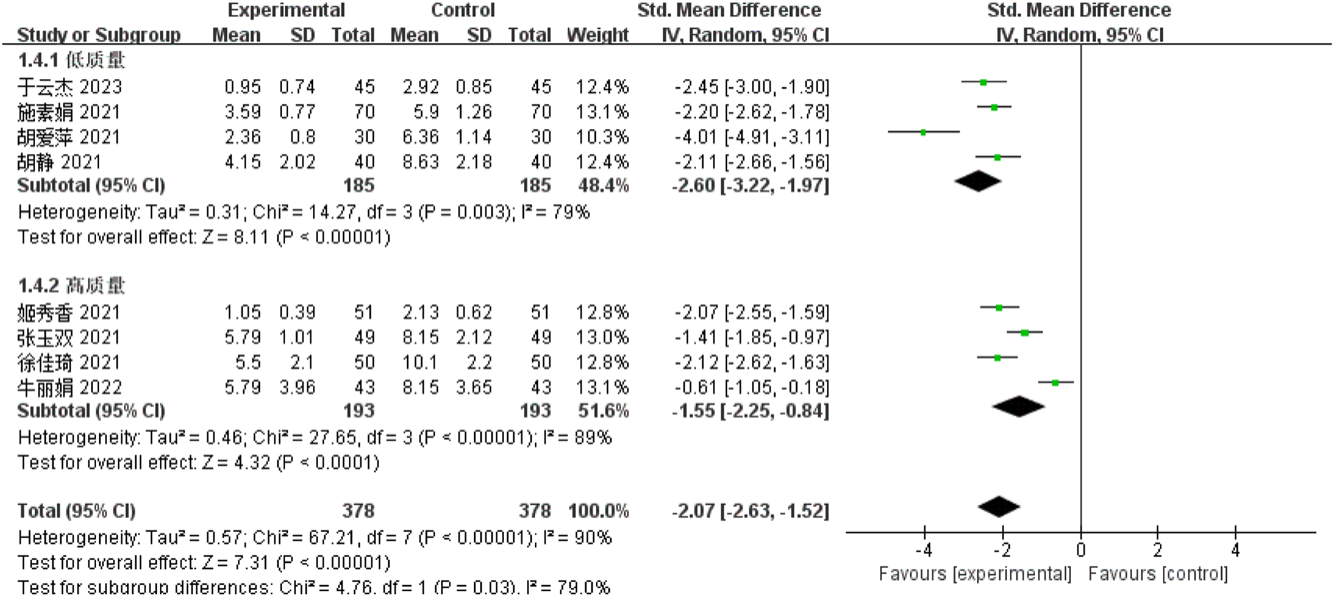
Forest map of ISI-Q-SF subgroup analysis.

Results of subgroup analysis: In order to investigate the heterogeneity of this outcome indicator, subgroup analysis was carried out according to the quality of the original literature. Four RCTs^[11–13,17]^ reported the effect of high-quality original literature biofeedback electrical stimulation on the ISI-Q-SF score of PSUI, and the results showed that the ISI-Q-SF score decreased after treatment. The performance of the observation group far surpassed that of the control group, with a statistically significant difference between them (SMD = −1.55, 95% CI = [−2.25 to −0.84], *P *< .01). Four RCTs^[8,14,19,20]^ disclosed the influence of low-quality original literature biofeedback electrical stimulation on the ISI-Q-SF score of PSUI. The results showed that the ISI-Q-SF scale score decreased after treatment, the observation group was markedly superior to the control group; the difference was statistically significant (SMD = −2.60. 95% CI [−3.22 to −1.97], *P* < .01.) Subgroup analysis based on the quality of the original literature indicated that the heterogeneity remained significant in both high-quality and low-quality original literature studies. Moreover, the quality of the original literature did not influence the ISI-Q-SF score of PSUI. Refer to Figure 7.

#### 3.4.4. The analysis of sensitivity

**Figure 8.**
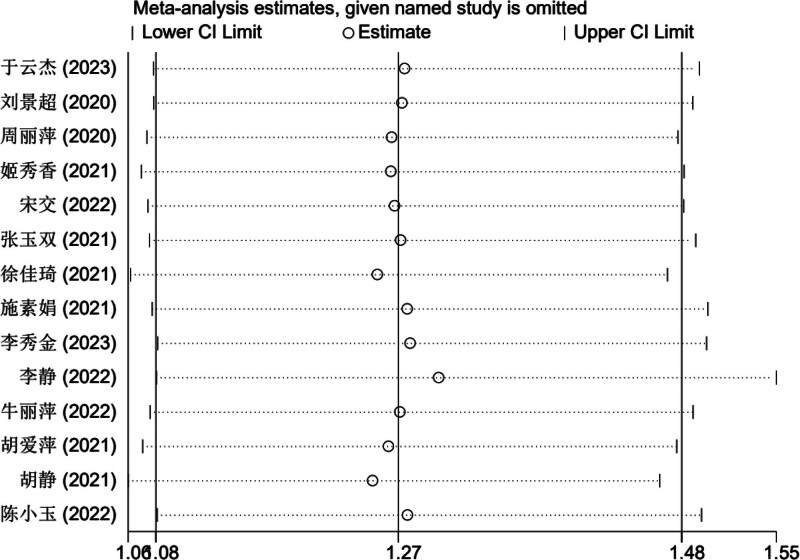
Sensitivity analysis diagram of included studies.

A meta-analysis was performed by eliminating individual references 1 at a time. By conducting a sensitivity analysis on the influence of a single study on the combined OR value, it was discovered that neither the OR nor the 95% confidence interval (95% CI) showed significant changes. Moreover, all the study point values were within the 95% CI of the final outcome. This indicated that removing any single item would not cause the result to exceed the confidence interval, suggesting that the meta-analysis findings of this study were robust (refer to Fig. 8).

#### 3.4.5. Publication bias

**Figure 9.**
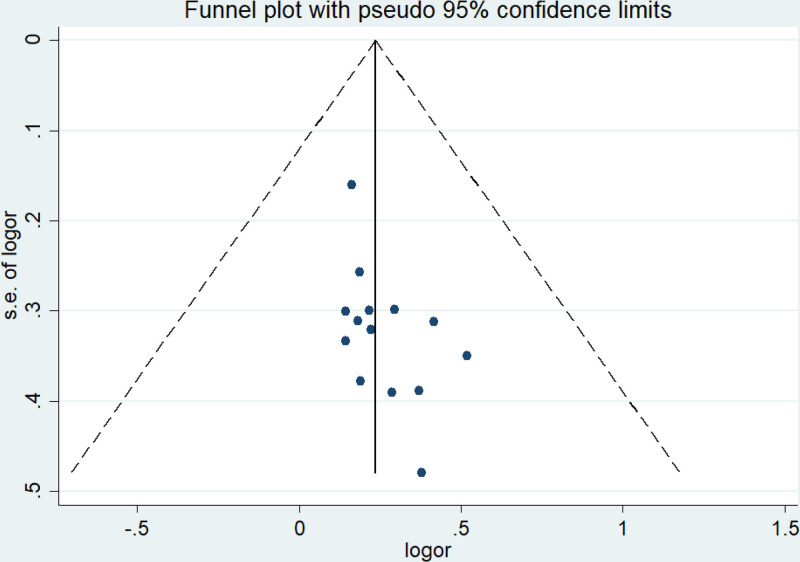
Funnel plot of publication bias in included studies.

**Figure 10.**
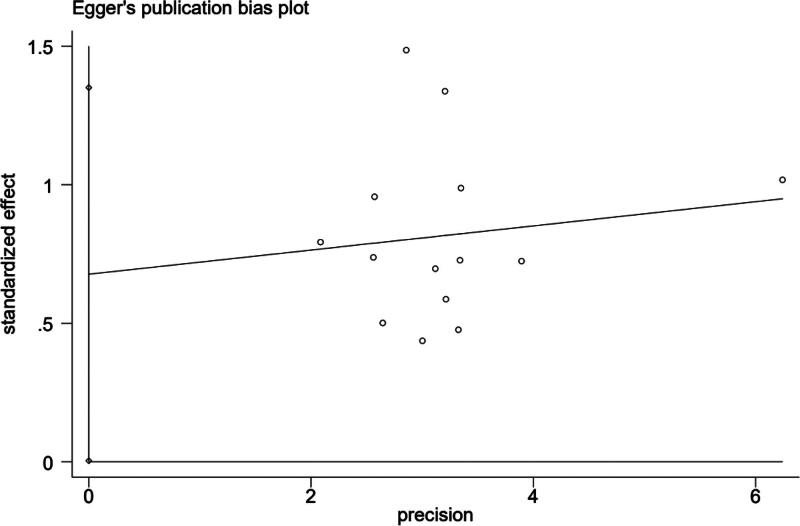
Egger test diagram included in the study.

**Figure 11.**
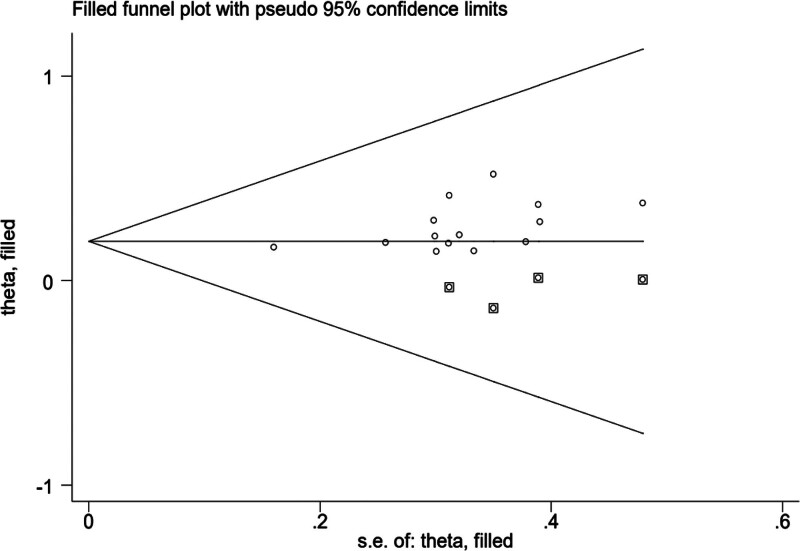
Schematic diagram of the shear compensation method included in the study.

Based on the clinical efficacy of the primary outcome index in the included literature, an analysis of publication bias was carried out. A funnel plot was constructed, with the logarithm of the OR for each study serving as the horizontal axis and the standard error of the logarithm of the OR as the vertical axis. As shown in Figure 9, due to the potential subjectivity in the evaluation of the funnel plot, Egger test was employed to further examine the symmetry of the funnel plot. The results indicated the presence of publication bias (Egger test, *t* = 2.19, *P* = .049 < .05), so it is necessary to use the scissor-supplement method to evaluate the stability of the combined results (see Figs. 10 and 11).

The results of shear and complement method show that: The results of the fixed-effect model and random effect model were reported simultaneously. For the heterogeneity test, *Q* = 3.230, *P* = .997. Using the random effect model, the combined result of effect indicators was logOR = 1.274, and the 95% confidence interval was (1.119, 1.429). After 4 iterations with Linear, the software estimated the number of missing studies, and the result was 4. Finally, after the data from 5 virtual studies were included, the meta-analysis of all studies was conducted again, and the results showed that the heterogeneity test was as follows: *Q* = 6.434, *P* = .983. The combined impact of the effect indicators was logOR = 3.404, and 95% CI was (2.944, 3.936) using the REM. The results of the 4 virtual studies were not reversed, so the combined results were robust.

## 4. Discussion

In recent years, fundamental studies on rehabilitating PSUI have gradually increased. Although some studies have developed some clinical rehabilitation treatment methods, unfortunately, the curative effect of transformation from basic research to clinical practice is not ideal, which is attributed to the fact that traditional clinical rehabilitation training is relatively simple. The sensory stimulation of patients is too little. Some patients still need help using it alone to obtain satisfactory curative effects. Therefore, seeking rehabilitation treatment methods with higher curative effects and fewer adverse reactions is essential.

This study included a total of 14 literature ^[8–21]^ for meta-analysis that used biofeedback electrical stimulation therapy, All included studies used clinical efficacy as the primary evaluation index, employed the Ingelman-Sundberg scale^[22]^ to assess treatment outcomes, and monitored the overall effective rate. Clinical effective rate is typically defined as the sum of the clinical recovery rate, significant efficacy rate, and effective rate in patients with PSUI. This indicator is widely used in modern TCM clinical research to comprehensively evaluate the overall treatment effectiveness.^[7]^ According to the results of the meta-analysis, biofeedback electrical stimulation shows significant therapeutic effects in treating PSUI. The 95% confidence interval of the overall effective rate lies to the left of the ineffective line, indicating a clear treatment effect. Furthermore, the difference in effect size reached statistical significance, further confirming the effectiveness of this treatment method in clinical applications. This result provides reliable evidence supporting biofeedback electrical stimulation as an effective therapeutic approach. Compared to conventional training alone, biofeedback electrical stimulation shows significant advantages in improving clinical efficacy, and its effect is clearly superior to that of patients receiving no treatment or rehabilitation treatment only. Therefore, clinical practice should emphasize the combined application of biofeedback and electrical stimulation, using effective joint therapy to further enhance treatment outcomes for patients.

Numerous literature^[9–11,14–16,18,21]^ used pelvic floor muscle strength as the outcome index. They adopted the international general perineal muscle strength detection method to evaluate patients’ pelvic floor muscle strength before and after an intervention. Compared to the control group, patients in the observation group showed significant advantages in both clinical efficacy and pelvic floor muscle strength, with a notable improvement in their pelvic floor muscle strength scores. This suggests that the treatment method in the observation group effectively enhances pelvic floor muscle function in patients. Yunjie and Xiu^[8]^ pointed out that biofeedback electrical stimulation can compensate for the lack of conventional pelvic floor muscle training that can not act on the deep. Biofeedback electrical stimulation is to place electrodes in the vagina and apply currents of different frequencies and pulse width to promote passive contraction and relaxation of pelvic floor muscles of patients, guide patients to control contraction and contraction of pelvic floor muscles autonomously, cultivate correct contraction habits, improve muscle strength of patients, improve urinary leakage caused by urinary incontinence, and achieve the purpose of restoring muscle strength.^[23,24]^ Biofeedback electrical stimulation has demonstrated significant efficacy in restoring pelvic floor muscle function, facilitating neural pathway reconstruction, and enhancing patients’ ability to perform daily self-care, playing a positive role in the overall rehabilitation process.^[25,26]^

Based on studies,^[27]^ about 1/3 of pregnant women are affected by stress incontinence, and postpartum stress incontinence has a significantly increased risk of persistent symptoms 12 to 15 years after delivery. Eight kinds of literature^[8,11–14,17,19,20]^ measured the therapeutic effect of biofeedback electrical stimulation on PSUI by comparing the ISI-Q-SF scoring scale. The ISI-Q-SF scoring scale in the observation group showed no statistically significant difference between the 2 groups before treatment, which were similar, and after treatment, the indexes in both groups showed a decline. Moreover, the decreasing trend of the observation group was significantly better than that of the control group (*P* < .05), suggesting that biofeedback electrical stimulation has a significant effect on PSUI and is better than only early pelvic floor muscle training, which is an essential basis for the prognosis of the disease. With the improvement of the patient’s muscle strength, the degree of urinary incontinence and the impact on quality of life can be reduced. Biofeedback electrical stimulation therapy mainly uses currents of different frequencies and powers to effectively stimulate the nerve tissue in the vaginal area of patients, thereby causing muscle contraction in the muscle tissue near the urethra, thereby enhancing the function of the muscle group in the pelvic floor muscle of patients, improving the muscle fiber strength and reaction speed, and further improving the disease symptoms more significantly.^[28]^ This research method provides an effective treatment approach for improving PSUI and has shown certain therapeutic effects in clinical practice. To further validate its clinical application value, future studies should expand the sample size and conduct large-scale basic or clinical research to analyze in-depth the regulatory effects and mechanisms of biofeedback battery stimulation on the nervous system in patients with PSUI.

Given the significant heterogeneity among literature involving the ISI-Q-SF rating scale, this study also conducted subgroup and sensitivity analyses to further validate the reliability and robustness of the results;Considering the potential subjectivity of the funnel plot, this study employed Egger test to further assess the symmetry of the funnel plot. The results show that publication bias exists (Egger test, *t* = 2.19, *P* = .049 < .05), so it is necessary to use the scissor-supplement method to evaluate the stability of the combined results. The results of the shear compensation method showed that: after 4 iterations with the Linear method, the software estimated the number of missing studies, and meta-analysis was performed on all studies again. The data results of the 4 virtual studies were not reversed, so the combined results were robust. Subgroup analysis results showed that the frequency, frequency, current intensity, time, type, dose, and frequency of biofeedback electrical stimulation in the observation group varied across the 8 studies related to the ISIS-Q-SF score, reflecting differences in the treatment frequencies used in each study,^[8,11–14,17,19,20]^ suggesting that the differences in intervention measures among patients in the observation group may be a significant factor contributing to the heterogeneity observed in the study. After sequentially removing the included studies, the combined analysis of the remaining literature showed no significant changes in the *I*^2^ value and *P*-value, indicating that the meta-analysis was not sensitive to the exclusion of individual studies, and the results demonstrated high robustness. Meanwhile, the quality of the included studies was evaluated, and the results showed that the ISI-Q-SF scoring scale was not significantly influenced by the quality of the articles. However, due to the specificity of the intervention methods, it was difficult to implement blinding for both practitioners and patients in clinical trials. In this study, the Chinese literature included did not implement blinding for the evaluators, which could lead to an overestimation of the rehabilitation effects and result in publication bias. Some studies have pointed out that patients in the observation group received both pelvic floor muscle rehabilitation training and drug therapy during treatment, so the potential impact of rehabilitation training on treatment outcomes cannot be ruled out. However, the frequency, frequency, current intensity, time, drug type, dose and drug frequency of biofeedback electrical stimulation included in the paper are different, and the inconsistency of frequency and frequency makes it difficult to directly compare the intervention intensity between different studies. Meanwhile, Baseline characteristics of patients (such as age, sex, severity of disease, comorbidity, etc) were also different in the study, and these factors may also increase heterogeneity, all of which can be sources of heterogeneity in the ISIS-Q-SF score. The above analysis can be the source of ISI-Q-SF rating scale heterogeneity. Previous studies have mainly relied on relatively small sample groups, which may have limited the generalisability and external validity of the findings. In contrast, this paper expanded the sample size and adopted a wider group of participants, thus improving the representativeness and reliability of the study conclusions. At the same time, the effect size of multiple relevant studies was analyzed and synthesized to reach a more robust conclusion. The calculation of the comprehensive effect size not only improves the universality of the study, but also makes the interpretation of the research results more scientific and reliable.

Although this meta-analysis was conducted by screening relevant literature strictly according to inclusion and exclusion criteria, there are still some limitations. Due to these factors, differences in the frequency of biofeedback electrical stimulation may lead to clinical and methodological heterogeneity, which in turn could affect the interpretation and reliability of the research results. Further studies are needed to explore the effects of different frequencies and frequencies on patients with PSUI to explore the best intervention time for the treatment of PSUI with biofeedback electrical stimulation so as further to improve the clinical efficacy of biofeedback electrical stimulation and provide a better frequency combination for patients with PSUI. Secondly,based on the literature search results, the studies included in this research primarily had samples from China, which may affect the generalizability of the findings. To reduce the bias potentially introduced by small sample studies, future research should consider conducting larger-scale, multi-center clinical trials to enhance the applicability and representativeness of the results. Existing studies mainly focus on evaluating the short-term effects of biofeedback electrical stimulation treatment, with a lack of long-term monitoring of disease progression. Therefore, future research should pay attention to the long-term efficacy of patients receiving biofeedback electrical stimulation treatment and strengthen long-term tracking and monitoring.

(I) Principle of Biofeedback Technology. Biofeedback is a therapeutic method that uses electronic instruments to convert physiological information such as heart rate, blood pressure, and muscle tension in the human body into visual or auditory signals that can be perceived by patients. This enables patients to understand their own physiological state and, through self-regulation training, learn to control these physiological responses. In the treatment of postpartum urinary incontinence, biofeedback is mainly used to help patients perceive and control the contraction of pelvic floor muscles. By placing electrodes in the vagina or rectum, the electrical activity of the pelvic floor muscles is detected and converted into intuitive images or sound feedback for the patient. Based on the feedback signals, the patient consciously adjusts the muscle contraction, gradually improving the muscle contraction ability and coordination.(II) Principle of Electrical Stimulation Treatment. Electrical stimulation uses currents of different frequencies, intensities, and waveforms to stimulate the pelvic floor muscles, causing passive muscle contractions, thereby enhancing muscle strength and improving muscle function. Electrical stimulation can directly excite the neuromuscular tissue, promote muscle blood circulation, increase the recruitment of muscle fibers, and improve muscle contraction ability. At the same time, electrical stimulation can also regulate nerve reflexes, enhance the tension of the urethral sphincter, and improve urinary control function. (III) Combined Treatment Procedure.1. Pretreatment Assessment Before starting biofeedback electrical stimulation treatment, a comprehensive assessment of the patient is required. First, inquire in detail about the patient’s medical history, including the mode of delivery, duration of labor, and whether there are episiotomy or laceration of the perineum. Second, conduct a pelvic floor muscle function assessment. Through methods such as digital examination, pelvic floor electromyography, or biofeedback instrument detection, understand the contraction strength, duration, fatigue degree, and other indicators of the pelvic floor muscles. In addition, it is also necessary to assess the type and severity of the patient’s urinary incontinence and the degree of impact on the quality of life.2. Setting of Treatment Parameters. According to the patient’s assessment results, personalized biofeedback electrical stimulation treatment parameters are set for them. The frequency of electrical stimulation is generally between 2 and 100 Hz, and the intensity should be within the patient’s tolerance range without causing pain. Usually, it starts from a lower intensity and gradually increases. The pulse width is generally 200 to 500 μs. The goal setting of biofeedback training is determined according to the patient’s initial pelvic floor muscle function. For example, for patients with weaker muscle strength, the initial goal can be set to be able to contract the muscles continuously for 3 to 5 seconds, with 10 to 15 repetitions as a group, and 3 to 4 groups for each treatment session. As the treatment progresses, the contraction time and number of repetitions are gradually increased.3. Treatment Process. The patient takes a comfortable position, generally a supine position with the knees bent and the legs abducted. Place the electrodes of the biofeedback electrical stimulation therapy instrument in the vagina or rectum, connect the instrument, and turn it on. The treatment process is divided into 2 stages: electrical stimulation and biofeedback training. In the electrical stimulation stage, the patient feels the passive contraction of the pelvic floor muscles caused by the electrical current stimulation, which lasts for 10 to 15 minutes each time. After the electrical stimulation ends, the biofeedback training stage begins. The patient tries to voluntarily contract the pelvic floor muscles according to the feedback signals provided by the instrument, making them synchronized with the feedback signals. The therapist guides the patient on the correct muscle contraction beside the patient, avoiding the involvement of other muscles such as the abdomen and buttocks. Each biofeedback training session lasts for 15 to 20 minutes. The entire treatment process lasts about 30 to 40 minutes each time, and it is carried out 2 to 3 times a week. A course of treatment generally consists of 10 to 15 sessions.4. Home Training. In addition to receiving biofeedback electrical stimulation treatment in the hospital, patients also need to carry out continuous pelvic floor muscle training at home to consolidate the treatment effect. The therapist will formulate a detailed home training plan for the patient, including training methods, frequency, and intensity. Patients can use a portable pelvic floor muscle trainer or perform simple Kegel exercises, such as contracting the anal and vaginal muscles, holding for 3 to 5 seconds, and then relaxing, repeating the exercise. Each training session lasts for 10 to 15 minutes, 2 to 3 times a day. At the same time, patients should also pay attention to avoiding actions that increase abdominal pressure in daily life, such as bending over for a long time and lifting heavy objects.5. Evaluation of treatment effect. After completing a course of treatment, it is necessary to evaluate the treatment effect of the patient. The evaluation method is similar to that before treatment, including pelvic floor muscle function assessment, improvement of urinary incontinence symptoms, and quality of life scoring. By comparing various indicators before and after treatment, the treatment effect is judged. If the patient’s symptoms are significantly improved, consolidation treatment can be continued, and the treatment frequency can be reduced; if the effect is not significant, it is necessary to reevaluate the patient’s situation and adjust the treatment plan.

Postpartum urinary incontinence is closely related to pelvic floor muscle weakness, and pelvic floor muscle weakness is one of the important causes of postpartum urinary incontinence. Biofeedback electrical stimulation, as an effective treatment method, helps patients perceive and control the contraction of pelvic floor muscles through biofeedback technology, and combines electrical stimulation to promote passive muscle contraction, enhance muscle strength, and improve urinary incontinence symptoms. During the implementation of the treatment process, personalized assessment and parameter setting should be carried out according to the specific situation of the patient, and attention should be paid to the cooperation of home training to improve the treatment effect and the quality of life of postpartum women. In the future, further in-depth research is still needed to optimize the treatment plan and provide better treatment options for more patients with postpartum urinary incontinence.

In conclusion, biofeedback electrical stimulation has a specific effect on treating PSUI. It plays a unique and important role in improving the quality of life and living conditions of patients with PSUI, primarily by significantly enhancing the contraction and relaxation abilities of the pelvic floor muscles. PSUI should receive the same attention as pelvic floor dysfunction caused by other lower urethral disorders, and it is important to begin intervention treatment at an early stage. Future studies will focus more on the therapeutic effects of different rehabilitation methods and other treatments for PSUI, in order to provide strong evidence for further exploration of the effects of biofeedback electrical stimulation on the treatment of PSUI, thus promoting its further application and dissemination.

## Acknowledgments

The authors would like to express their thanks to the researchers and participants for their contributions to this article.

## Author contributions

**Conceptualization:** Jia-Yu Liu.

**Formal analysis:** Jia-Yu Liu.

**Funding acquisition:** Jia-Yu Liu, Lu-Wen Zhu.

**Investigation:** Bin-Han Wang.

**Methodology:** Bin-Han Wang, Lu-Wen Zhu.

**Project administration:** Lu-Wen Zhu.

**Resources:** Bin-Han Wang.

**Software:** Lu-Wen Zhu.

**Supervision:** Ji-Guang Pan.

**Validation:** Ji-Guang Pan.

**Visualization:** Ji-Guang Pan.

## References

[1] Jaffar A Mohd–Sidik S Nien FC. Urinary incontinence and its association with pelvic floor muscle exercise among pregnant women attending a primary care clinic in Selangor, Malaysia. PLoS One. 2020;15:e0236140. 32667936 10.1371/journal.pone.0236140 PMC7363082

[2] Zhu D Xia Z Yang Z. Effectiveness of physiotherapy for lower urinary tract symptoms in postpartum women: systematic review and meta-analysis. Int Urogynecol J. 2022;33:507–21. 34302516 10.1007/s00192-021-04939-z

[3] Nygaard IE Wolpern A Bardsley T Egger MJ Shaw JM. Early postpartum physical activity and pelvic floor support and symptoms 1 year postpartum. Am J Obstet Gynecol. 2021;224:193.e1–193.e19. 10.1016/j.ajog.2020.08.033 PMC7855223 32798462

[4] Myer ENB Roem JL Lovejoy DA. Longitudinal changes in pelvic floor muscle strength among parous women. Am J Obstet Gynecol. 2018;219:482. 10.1016/j.ajog.2018.06.003 PMC6436096 29902445

[5] Peng Y Miller BD Boone TB Zhang Y. Modern theories of pelvic floor support: a topical review of modern studies on structural and functional pelvic floor support from medical imaging, computational modeling, and electromyographic perspectives. Curr Urol Rep. 2018;19:9. 29435856 10.1007/s11934-018-0752-9

[6] Chinese Society of Obstetrics and Gynecology. Guidelines for diagnosis and treatment of female stress urinary incontinence (2017). Chin J Obstet Gynecol. 2017;52:289–93. in Chinese.

[7] Gu S Xu Y Zhao M. Effect of biofeedback electrical stimulation combined with pelvic floor functional training on postpartum stress urinary incontinence in primiparas. Med J Chin People Health. 2020;32:74–6.

[8] Yunjie Y Xiu H. Effect of biofeedback electrical stimulation combined with pelvic floor muscle training on postpartum stress urinary incontinence. Heilongjiang Med. 2023;4:675–8.

[9] Liu J Danfeng Li Yuntao H. Effect of biofeedback electrical stimulation combined with pelvic floor muscle training on postpartum stress urinary incontinence. Chin Community Phys. 20;36:44–5. in Chinese.

[10] Zhou L. Efficacy analysis of biofeedback electrical stimulation in the treatment of stress urinary incontinence. J Clin Med Lit Electron. 2019;7:49–50. in Chinese.

[11] Song J Hao Z Yangyang L. Effect of biofeedback electrical stimulation combined with routine pelvic floor rehabilitation training on stress urinary incontinence. Chin Sci Technol J Database Med Health. 2022;10:283–6.

[12] Zhang Y Yahong CAI Chunyan C. Effect of myoelectric biofeedback electrical stimulation combined with Kegel training on clinical efficacy and urodynamic indexes of postpartum patients with stress urinary incontinence. Heilongjiang Traditional Chin Med. 201;50:112–4.

[13] Jiaqi X Xiexing W Jin Z. Effect of biofeedback combined with electrical stimulation in the treatment of postpartum stress urinary incontinence. China Maternal Child Health Care. 21;36:279–82.

[14] Shi S Xiaoying Y Yinfen M Shuijuan Z. Clinical observation of early rehabilitation training combined with electrical stimulation biofeedback in the treatment of postpartum stress urinary incontinence. China Maternal Child Health Care. 21;36:2469–72.

[15] Xiujin Li Zhiying Y Yiping C. Effect of pelvic floor muscle electrical stimulation biofeedback combined with Kegel training on pelvic floor muscle strength and stress urinary incontinence in postpartum patients with pelvic floor dysfunction. Chin Med Innov. 2019;20:159–62.

[16] Li J. Effect of biofeedback electrical stimulation combined with pelvic floor muscle training on postpartum stress urinary incontinence. J Mod Electrophysiol. 202;29:98–101.

[17] Niu L. Clinical effect of myoelectric biofeedback electrical stimulation combined with Kegel training on postpartum stress urinary incontinence. Chin Sci Technol J Database Med Health. 2022;11:0033–5.

[18] Wang C. Effects of biofeedback electrical stimulation combined with vaginal dumbbell training on urinary dynamics in postpartum patients with stress urinary incontinence. Reflexol Rehabil Med. 2012;2:152–5.

[19] Aiping H Yuhong Z. Application of pelvic floor biofeedback electrical stimulation in the treatment of stress urinary incontinence. J Anhui Health Vocational Tech Coll. 2021;20:134–135,137. in Chinese.

[20] Jing H Yuning Y Si C. Effect of Kegel training combined with biofeedback electrostimulation on postpartum stress urinary incontinence. J Xuzhou Med Univ. 2021;41:464–8.

[21] Chen X. Effect of biofeedback electrical stimulation and pelvic floor muscle training on postpartum stress urinary incontinence. Chin J Health Stand Manage. 2012;13:50–2.

[22] Zhu L Sun ZJ. Guidelines for diagnosis and treatment of female stress urinary incontinence (2017). Chin J Obstet Gynecol. 2017;52:289–93. 10.3760/cma.j.issn.0529-567X.2017.05.001 28545265

[23] Sahin N Yesil H Gorcan B. The effect of pelvic floor exercises performed with EMG biofeedback or a vaginal cone on incontinence severity, pelvic floor muscle strength, and quality of life in women with stress urinary incontinence: a randomized, 6-month follow-up study. Int Urogynecol J. 2022;33:2773–9. 35028701 10.1007/s00192-021-05006-3

[24] Jacobsen LV Jørgensen CS Sørensen KM K. The efficacy of physiotherapeutic intervention with biofeedback assisted pelvic floor muscle training in children with dysfunctional voiding. J Pediatr Urol. 2021;17:793.e1–6. 10.1016/j.jpurol.2021.09.022 34635441

[25] Alouini S Memic S Couillandre A. Pelvic floor muscle training for urinary incontinence with or without biofeedback or electrostimulation in women: a systematic review. Int J Environ Res Public Health. 2022;19:2789. 35270480 10.3390/ijerph19052789 PMC8910078

[26] Li Z Xu T Li Z Gong J Liu Q Zhu L. Lower urinary tract symptoms 7 years after the first delivery: correlation to the mode of delivery. Neurourol Urodyn. 2019;38:793–800. 30644569 10.1002/nau.23922

[27] Wang X Xu X Luo J Chen Z Feng S. Effect of app-based audio guidance pelvic floor muscle training on treatment of stress urinary incontinence in primiparas: a randomized controlled trial. Int J Nurs Stud. 2020;104:103527. 32058140 10.1016/j.ijnurstu.2020.103527

[28] Zou L Gao L. Effect of biofeedback instrument combined with pelvic floor muscle functional exercise on postpartum stress urinary incontinence patients. J Qilu Nurs. 2020;26:52–4.

